# Comparative Ecophysiological Study of Salt Stress for Wild and Cultivated Soybean Species from the Yellow River Delta, China

**DOI:** 10.1155/2014/651745

**Published:** 2014-05-25

**Authors:** Gang Wu, Zhengda Zhou, Peng Chen, Xiaoli Tang, Hongbo Shao, Hongyan Wang

**Affiliations:** ^1^State Key Laboratory of Urban and Regional Ecology, Research Center for Eco-Environmental Sciences, Chinese Academy of Sciences, Beijing 100085, China; ^2^The Graduate University of Chinese Academy of Sciences, Beijing 100049, China; ^3^Key Laboratory of Coastal Biology & Bioresources Utilization, Yantai Institute of Coastal Zone Research (YIC), Chinese Academy of Sciences (CAS), Yantai 264003, China; ^4^Institute for Life Science, Qingdao University of Science & Technology (QUST), Qingdao 266042, China

## Abstract

Osmotic and ionic stresses were the primary and instant damage produced by salt stress. They can also bring about other secondary stresses. Soybean is an important economic crop and the wild soybean aroused increasing attention for its excellent performance in salt resistance. For this reason, we compared the different performances of *Glycine max* L. (ZH13) and *Glycine soja* L. (BB52) in both young and mature seedlings, hoping to clarify the specific reasons. Our research revealed that, compared to the cultivated soybean, the wild soybean was able to maintain higher water potential and relative water content (RWC), accumulate more amount of proline and glycine betaine, reduce the contents of Na^+^ and Cl^−^ by faster efflux, and cut down the efflux of the K^+^ as well as keep higher K^+^/Na^+^ ratio. And what is more is that, almost all the excel behaviors became particularly obvious under higher NaCl concentration (300 mM). Therefore, according to all the detections and comparisons, we concluded that the wild soybean had different tolerance mechanisms and better salt resistance. It should be used as eminent germplasm resource to enhance the resistant ability of cultivated soybean or even other crops.

## 1. Introduction

The adverse environments which can bring about disastrous damage to plants are multiple, including drought, salinity, mineral deficiency, and extreme temperature [1]. Scientists throughout the world spare no efforts to study and assess the influences of all the stresses on plants all the time, and a large number of discoveries have been acquired. However, the effects of the salinity on plants attract more attentions than others [2, 3]. The adverse impacts imposed by salt stress are osmotic stress, ionic stress, nutrient imbalance, and the production of reactive oxygen species; then, the plants would display declining growth and photosynthesis rate, even death in the end [4]. Among all the influences, the osmotic and ionic stresses are the dominating and fundamental ones. The osmotic stress is due to the high concentration of the salinity in the soil which hampers the roots to absorb water from the soil. And the ionic stress is because of the high concentration of salts within the plants [5].

As is well known, the osmotic stress is always accompanied with drought stress under which physiological drought often occurs, yet it can also be induced by salt stress [6]. Under salt stress, the water potential of the soil is reduced by the large quantity of ions, so it is very difficult for the roots to take up enough water to maintain natural physical development [7]. Under this circumstance, the stomatal conductance reduced immediately and transiently [8]. Moreover, related researches on halophyte such as* R. mucronata*,* Urochondra setulosa*,* and S. salsa *have demonstrated that the water potential and transpiration rate reduced along with the increasing salt concentration [9–11]. To accommodate the water balance, plants can also reduce the loss of the water and lower water potential through the accumulation of the compatible solutes. The compatible solutes are organic and low molecular metabolites, and, at the same time, they do not harass the normal cellular order [12]. The common and effective compatible solutes include proline, glycine betaine, polyols, fructans, trehalose, sucrose, and mannitol [4, 12, 13]. In addition, genes of many osmolytes have been identified, cloned, and introduced into plants, producing an immense number of transgenic plants with enhanced resistance.

With a great deal of ions mainly Na^+^ and Cl^−^ entering into intracellular, the ion homeostasis of the cell is disturbed, manifested in high concentration of Na^+^ and Cl^−^, alterations in Na^+^/K^+^ rate, and disordered metabolism [14]. The Na^+^ is able to compete with K^+^ to bind to the plasma membrane obstructing the sorption of K^+^, which plays very important roles in protein synthesis, cofactor of many enzymes, osmotic adjustment, and cytosolic ion homeostasis [15]. It has been reported that the replacement of the K^+^ by Na^+^ can lead to a forfeit of the chloroplast functions bringing about the wild loss of water [16]. The good news is that the plants have acquired a plethora of different mechanisms to adapt to the salt stress. Among them, control of the ion exchange and xylem loading is the key factor [5]; moreover, maintaining a high activity of H^+^-ATPase is considered another efficient measure [17]. For instance, the H^+^-ATPase in plasma membrane is able to influence the transport of the essential ions by keeping the indispensable electrochemical gradient (Δ*μ*H) [18]. The noxious ions can be discharged from the cells or be compartmentalized into the organelles by the ion transporters which are supported by the H^+^-ATPase. Now, the plasma membrane Na^+^/H^+^ exchangers (SOS1) and the tonoplast ones (NHX) are the most important objects and the transgenic engineering focusing on them has acquired tremendous achievements. Particularly, it is worth mentioning that the signal transduction in the regulation of ion concentration and homeostasis has also qualified its irreplaceable positions, the most well known of them is the discovery of the SOS signal transduction.

As mentioned above, salinity is the key factor which limits the growth and production of the plants. On the one hand, salinity is an ever-present stress to the plants especially to the crop yields, and, on the other hand, the salinization is becoming increasingly serious as a result of human activities [3, 19]. Thus developing crops with more tolerance to the salt stress is imperative and helpful [20]. The breeders throughout the world are striving to screen some cultivated crops or their wild relatives to acquire the salt tolerance species [21]. Owing to the high oil and protein content in its seeds, soybean is an important economic dicot crop [22–24]. And, above all, the demand for soybean, particularly in our country, is increasing continuously [25]. However, as a salt-sensitive species, the growth and development of soybean are severely affected by salt stress [26, 27]. Exploiting resistant varieties and improving salt tolerance of soybean, therefore, became the goal of many researchers [28].

Almost all the crops are sensitive to salinity, and soybean is no exception [29]. Soybean germplasms show up multifarious salt tolerance capability and the different extents of the damage among the different genotypes also indicate the genetic variability in salt resistance [30]. The wild soybean,* Glycine soja, *is regarded as the progenitor of the cultivated soybean,* Glycine max*. Although the domestication of soybean has endowed the cultivated soybean with many advantages in morphological and physiological traits [31], studies have revealed that the wild soybean had more genetic diversity especially in stress resistance. Exploring and importing excellent genes from wild species offered an effective and fast way to facilitate the agricultural development [32]. Many studies have also demonstrated that the related wild relatives of crops have the necessary traits to improve the cultivated species [33]. In addition, several agricultural traits of wild soybean have already been introduced into cultivated soybean [34, 35]. So the wild soybean may act as a source for enhancing the tolerance of the cultivated ones to boost the output under salt stress [36].

In this study, two genotypes of soybean,* Glycine max* L. (ZH13) and* Glycine soja* L. (BB52), were used to discuss the different responses to osmotic stress and ionic stress entrusted by salts. They share the same gene pool and researches have suggested that they can be hybridized with fertile offspring [37]. Thus, we expected that more and better properties, which can be applied to improve the germplasm resource, would be unearthed through the comparison between* Glycine max* L. and* Glycine soja* L. Ultimately, we found that the* Glycine soja* L. indeed is superior to* Glycine max* L. in salt tolerance reflected by a series of indexes and can be used as the donor for cultivated soybean resistant improvement.

## 2. Materials and Methods

### 2.1. Plant Materials, Growth Conditions, and Treatments

The ecotypes of the soybean adopted in this study were salt-sensitive* G. max* ZH13 cultivar and the salt-tolerant* G. soja* BB52. The ZH13 was widely cultivated in China and provided by Shandong Academy of Agricultural Sciences, while the BB52 was collected from Yellow River Delta, Shandong Province, China. The homogeneous, full, and unbroken seeds were collected and sewn in medium for germination. The medium was laid with two layers of filter paper which were moistened with Hoagland nutrient solution. Firstly, the seeds were fostered at 25°C for 5 d in the darkness and then the young seedlings were transferred to the plastic pots filled with vermiculite for further development in artificial climatic chambers (Huier, China). The plants were cultivated under 12 h light/12 h dark with 25/18°C, respectively. The humidity of the climatic chambers was kept at 65%.

The salt treatments with different NaCl dosages were applied to the plants in seedling stage and mature period, respectively. Plants with uniform growth pattern were selected for treatments. The salt treatments were carried out by irrigating Hoagland solution including 0, 50, 100, 200, or 300 mM/L NaCl and sustained for 7 d. The higher NaCl concentrations (>50 mM) were imposed incrementally to the final concentration by 50 mM step every day. The samples were harvested after treatments and preserved in −80°C for measurement. Each treatment had three biological replications at least.

### 2.2. Measurement of Water Potential (*ψ*_w_)

The instrument used in this experiment was WP4-T Dewpoint PotentiaMeter (USA) and the principle of the measurement was chilled-mirror Dewpoint technique. The leaf and root samples were sipped up and placed in a sample cup. It should be noticed that the sample cup should be covered completely.

### 2.3. Measurement of Relative Water Content (RWC)

The leaf and root samples were harvested and weighed (fresh weight, FW). Then the samples were soaked in deionized water at 4°C for 24 h and weighed (saturated fresh weight, SFW) after being blotted up. Finally, the samples were killed out at 105°C for 30 min and stovinged at 80°C for 48 h. The remaining weight was the dry weight, DW. RWC was calculated as RWC = (FW−DW)/(SFW−DW). The specific procedures of the RMC measurement were referred to by Flexas et al. [38].

### 2.4. Proline Content Determination

Proline content was measured according to Bates et al. [39] with a little modification. Samples were ground and homogenized in 3 mL 5% (w/v) sulphosalicylic acid extracting for 10 min at 100°C. The homogenate was centrifuged at 13,000 g for 10 min, and then the supernatant was collected as extracting solution. 2 mL glacial acetic acid and 3 mL ninhydrin reagent were added to 2 mL of the supernatant and incubated at 100°C for 40 min. After cooling to room temperature, 5 mL toluene was added to the reaction liquid and the mixed liquor would layer; the red material was extracted into the toluene phase. The absorbance of the toluene phase was measured at 520 nm at last. The standard curve was plotted according to the proline solution of known concentration.

### 2.5. Measurement of Glycine Betaine Content

The measurement of the glycine betaine was assayed according to Gorham et al. [40]. Briefly, the plant tissues were homogenized in 3 mL methanol-chloroform-KHCO_3_ solution containing methanol : chloroform : 0.2 mM KHCO_3_ = 12 : 5 : 1. The homogenate was incubated and shaken at 60°C for 20 min and centrifuged at 10,000 g for 10 min after cooling to 4°C. The supernatant liquid was collected in a new centrifuge tube and the precipitate was washed again and again. It was washed with the same extract solution at first and then was washed with methanol-H_2_O (1 : 1) solution. All the supernatant was transferred to the above tube and 2 mL chloroform and 3 mL distilled water were added. The mixed liquor was vortexed and centrifuged at 10,000 g for 10 min. Then the supernatant was the crude extract of glycine betaine. The crude extract was purified further by passing through ion exchange column and distillation subsequently. In addition, the residue was dissolved by methyl alcohol and filtered through Millipore filter. The content of the glycine betaine was measured by HPLC (high performance liquid chromatograph) at 195 nm. The value was obtained according to the standard curve prepared with pure glycine betaine (Sigma, USA) solutions.

### 2.6. Measurement of Na^+^, K^+^, and Cl^−^ Content

The extraction and measurement of Na^+^, K^+^, and Cl^−^ were performed according to Yan et al. [41]. The samples were killed out at 105°C for 30 min and baked at 85°C to standing weight. The dried material was smashed and sifted into powder. 100 mg powder was added to 15 mL of deionized H_2_O and incubated in boiling water for 2 h. The extracting solution was centrifuged at 10,000 g for 20 min, and then the supernatant was used for the measurement of the ion content. The contents of Na^+^ and K^+^ were detected by atomic absorption spectrophotometer (PAS-990, PERSEE, China), while the Cl^−^ was analyzed through a Cl^−^ electrode (Leici, China). The amounts of ions were calculated according to the standard curve prepared with pure NaCl (for Na^+^ and Cl^−^) and KCl (for K^+^) solutions.

### 2.7. Measurements of Net Na^+^, H^+^, and Cl^−^ Fluxes

The net fluxes of Na^+^, K^+^, H^+^, and Cl^−^ were carried out by noninvasive microtest technique (NMT-YG-100, Younger, USA). The principle of the measurement was that the ion fluxes were calculated from the ion specific PD (potential difference) between two points.

The glass micropipettes which were prepulled and silanized in advance were filled with a backfilling solution (Na^+^: 100 mM NaCl; K^+^ : 100 mM KCl; H^+^: 40 mM KH_2_PO_4_ + 15 mM NaCl; Cl^−^: 100 mM KCl) to a length of approximately 1 cm. Then the micropipettes were filled with selective liquid ion exchange cocktails and an Ag/AgCl wire electrode holder was inserted in the micropipettes.

Ion-selective electrodes were firstly calibrated before the flux measurements. The concentrations of the calibrator were as follows: (1) Na^+^: 5 mM, 2 mM, and 1 mM (2 mM in measuring solution); (2) K^+^: 1 mM, 0.5 mM, and 0.1 mM (0.5 mM in measuring solution); (3) H^+^: pH 5, 6, and 7 (6 in measuring solution); (4) Cl^−^: 2 mM, 0.5 mM, and 0.25 mM (0.5 mM in measuring solution). Only electrodes with Nernstian slopes >50 mV/decade (<−50 mV/decade for Cl^−^ electrodes) were used.

The root apexes at the length of 3 cm were fastened to the bottom of measuring dish, washed with the deionized water, and incubated immediately in the measuring solution for 20 min. The measuring sites were located at 500 µm from the root apex. The measuring solution was as follows: (1) Na^+^: 0.1 mM KCl, 0.1 mM CaCl_2_, 0.1 mM MgCl_2_, 2 mM NaCl, and 0.3 mM MES, pH 6.0, adjusted with choline and HCl; (2) K^+^: 0.1 mM KCl, 0.1 mM CaCl_2_, 0.1 mM MgCl_2_, and 0.3 mM MES, pH 6.0, adjusted with choline and HCl; (3) H^+^: 0.1 mM KCl, 0.1 mM CaCl_2_, 0.1 mM MgCl_2_, and 0.3 mM MES, pH 6.0, adjusted with NaOH and HCl; (4) Cl^−^: 0.05 mM KCl, 0.05 mM CaCl_2_, 0.05 mM MgCl_2_, and 0.25 mM NaCl, pH 6.0, adjusted with choline and H_3_PO_3_. Ultimately, the microvolts differences were converted into net ion fluxes with the help of the software JCal V3.0 (Xuyue Sci. and Tech., China).

### 2.8. Statistical Analysis

All the data in this study were analyzed by Microsoft Excel 2007, and, moreover, the one-way ANOVA was performed by adopting SPSS computer package (SPSS Inc., USA). Every data acquired in the experiments is the mean of three biological replications at least. Significant differences between means were determined through LSD test. Differences were considered statistically significant when *P* < 0.05. All data were presented as mean ± SD. In addition, the figures were all created with SigmaPlot 10.0 (Systat Software, Inc., Germany).

## 3. Results

### 3.1. The Effects of NaCl on Water Potential and Relative Water Content (RWC)

**Figure 1:**
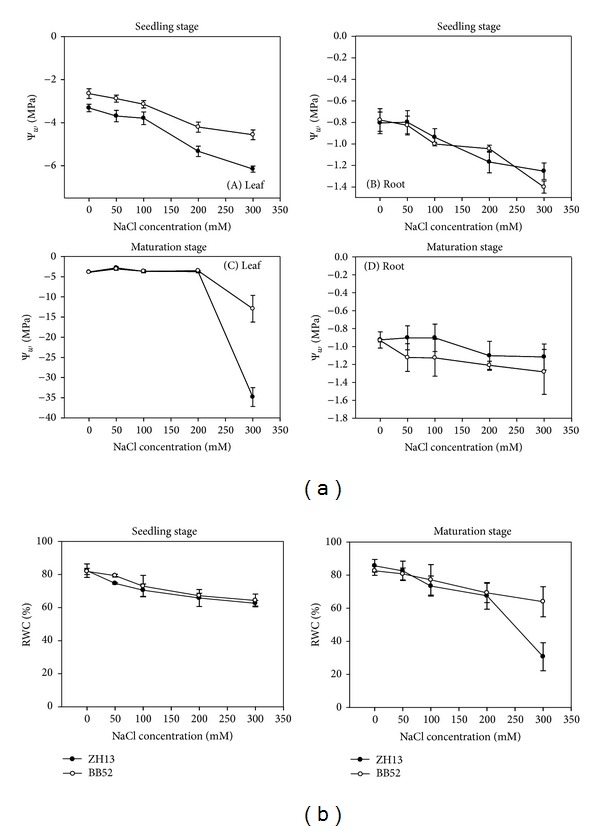
Changes of water potential (*ψ*_w_, A) and relative water content (RWC, B) under salt stress.

Just as we mentioned above, the water balance of the whole plant would be disturbed primarily whether under salt or drought stresses. The high concentration of ions in the soil solution lowered its water potential; thus it became more difficult for plants to absorb enough water. To determine the different influences of salts on water balance of two types of soybean, the *ψ*_w_ and RWC of ZH13 and BB52 were assayed after salt treatment. The young seedlings and the mature seedlings were detected, respectively. From Figures 1(a) and 1(b), we can clearly see that, with the increasing salt concentration, the *ψ*_w_ and RMC were increasingly severely reduced, especially for mature seedlings of ZH13 (Figure 1(a)). The *ψ*_w_ of the leaves of mature ZH13 was reduced 11.2 times under 300 mM treatment, and the RWC of the mature ZH13 was lessened by 64.2%. Under 200 mM NaCl treatment, there were 66.7% of the ZH13 at mature stage displayed wither and fall which were the classical symptom of hydropenia, while all the plants put out the classical symptom of hydropenia under 300 mM NaCl treatment and more than 30% have already been dead. For the RWC, there was no obvious difference between them in young seedlings, while in mature seedlings the BB52 had higher RWC, especially under 300 mM NaCl treatment (Figure 1(b)). Compared to ZH13, though the *ψ*_w_ and RMC of the wild soybean BB52 were also downregulated, the degree of the declining was quite small. Most of them were able to maintain development under 300 mM NaCl treatment. In addition, although the *ψ*_w_ and RMC of the young seedlings were all affected seriously, both types can manage to survive and few of them became withered.

### 3.2. The Effects of Salt Treatment on Proline and Glycine Betaine Content

**Figure 2:**
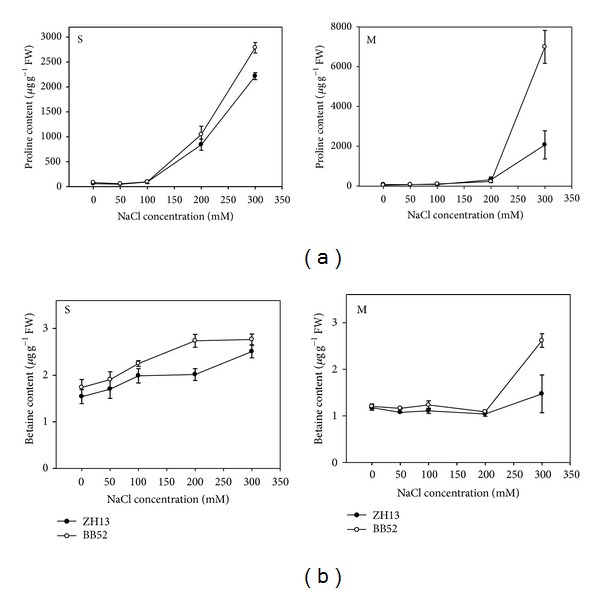
Effects of salt stress on proline (a) and glycine betaine (b) accumulation. S stands for seedlings and M stands for mature seedling in the picture.

Proline is one of the most important compatible solutes. On the one hand, its accumulation in the cells of plants is able to lower the water potential of the cell to maintain the hydrologic balance under abiotic stress, and, on the other hand, the proline acts as molecular chaperone to stabilize the protein conformation by binding to the proteins [42]. Thus, to some degree, the content of the proline reflects the resistant ability of the plants.  Figure 2(a) shows that the contents of the proline were almost identical in two types of soybean under normal conditions, while with increasing salt concentration, the content of proline was also obviously increased. For young seedlings, the content of the proline began to augment rapidly when the concentration of NaCl was above 100 mM, and the wild soybean BB52 was shown to be more outstanding. Under 300 mM NaCl treatment, the rates of increase of proline contents in young seedlings of ZH13 and BB52 were able to reach 34.2 and 38.2, respectively, yet the extents of the augment for mature seedlings were more sharp attaining 24.9 and 151.1 times, respectively (Figure 2(a)).

Glycine betaine is another important compatible solute with almost the same functions as those of proline; therefore, we also measured its changes during the salt stress. Its content showed the same trend as proline. For the young seedlings of ZH13 and BB52, the contents of the glycine betaine were increased by 59.4% and 62.9% under 300 mM NaCl treatment, respectively (Figure 2(b)). The changes were more obvious in mature seedlings of ZH13 and BB52 and they were 25.1% and117.2% (Figure 2(b)). The pronounced difference also told us that the wild soybean possessed better abilities of osmotic adjustment.

### 3.3. The Fluctuation of the Ion Contents under Salt Stress

**Figure 3:**
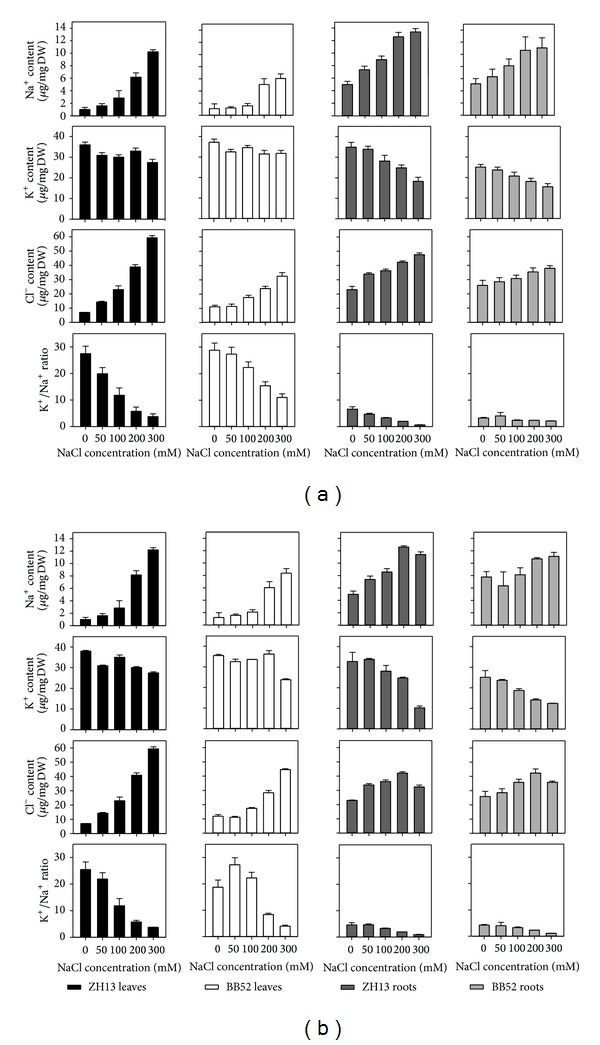
(a) Changes of ion content in young seeding under salt stress. (b) Changes of ion content in mature seeding under salt stress.

The difference in ion concentration is another important feature under salt stress; thus, we also detected the contents of the Na^+^, K^+^, and Cl^−^ under different NaCl treatments. Firstly the situation changes of young seedlings were examined. From Figure 3(a), we can find that the contents of both Na^+^ and Cl^−^ of leaves and roots increased significantly with the increase of NaCl concentration, manifesting a similar trend. With 300 mM NaCl treatment, the content of Na^+^ in leaves and roots of ZH13 increased 10.2 and 2.8, respectively; the situation of Cl^−^ was 8.5 and 2.1. However, the values for BB52 were 5.5, 2.1, 2.9, and 1.5, correspondingly. Between two soybean strains, the variation of ion contents in roots was tiny, but the difference was great in leaves (Figure 3(a)). For K^+^, the situation was exactly adverse. The consequence shows that the NaCl inhibited the absorption of K^+^ significantly; thus, the K^+^ content decreased in leaves and roots, especially in roots. K^+^ has a key role in the growth and development of plants; hence, the K^+^/Na^+^ ratio is a marker of the resistance. Generally, compared to ZH13, the degree of change of all the ion contents in BB52 was more at ease, the content of K^+^ in specific. In other words, under salt stress, the BB52 maintained a significantly higher K^+^/Na^+^ ratio than ZH13, just as the result suggests. Therefore, we can conclude that the BB52 was endowed with stronger resistance. Almost the same trends were discovered in mature seedlings (Figure 3(b)). The only difference was that the distinguished extent of the K^+^/Na^+^ ratio lessened. In addition, the content of the Na^+^ and Cl^−^ contents were abound in senile leaves.

### 3.4. The Behavior of Ion Flux under Salt Stress

**Figure 4:**
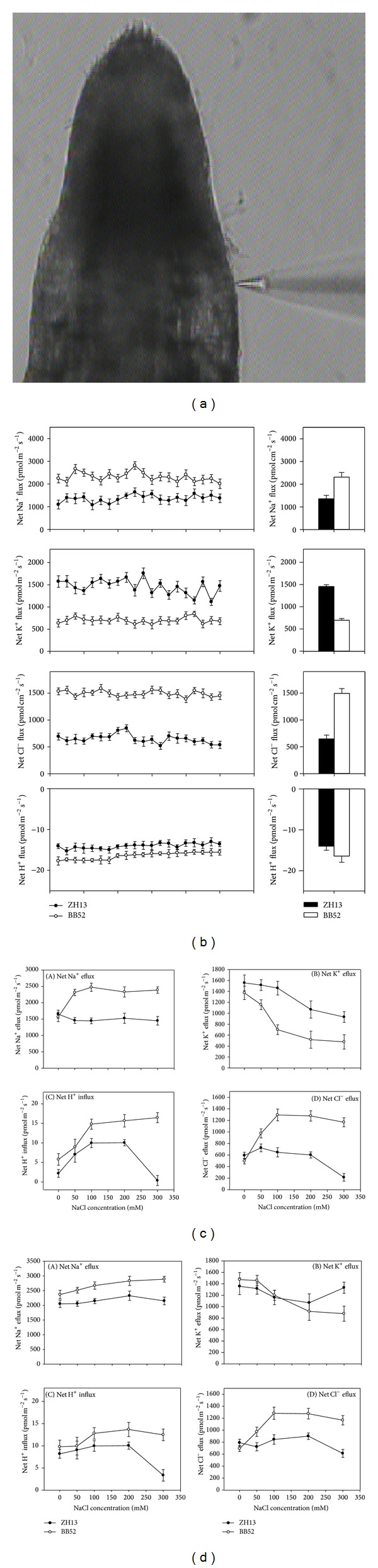
(a) Schematic diagram of noninvasive microtest technique (NMT); (b) typical flux pattern of Na^+^, K^+^, H^+^, and Cl^−^; (c) the situation of ion fluxes in young seeding under salt stress; (d) the situation of ion fluxes in mature seedling under salt stress.

In this experiment we adopted noninvasive microtest technique (NMT) (Figure 4(a)) to test the ion flux. Firstly, the typical flux patterns of Na^+^, K^+^, H^+^, and Cl^−^ were detected and the specific situation was displayed in Figure 4(b). The typical figures tell us that the Na^+^, K^+^, and Cl^−^ were excluded from the cells of roots, while the H^+^ was shifted into the cells. The experimental results (Figure 4(c)) of young seedling show that, with increasing salt concentration, the Na^+^ effluxes of BB52 were markedly increased and the maximum, 52.4%, was reached at 300 mM NaCl. Yet the ZH13 suffered the same stress and did not display significant efflux (*P* > 0.05). The effluxes of K^+^ were decreased with the increased NaCl; the content was dropped to 65.2% in wild soybean exceeding 40.1% in cultivated one. Moreover, there was visible difference in the absorption of H^+^ between them. The influx of H^+^ was increased constantly in wild soybean reaching 185.3% under 300 mM treatment, but the situation was not suitable for ZH13. Although the ion flux was also quickened in ZH13 under 0–200 mM treatment, the flux declined sharply in 300 mM NaCl, almost reaching 0. Besides, the flux of Cl^−^ was also different. Under 300 mM NaCl, the efflux of Cl^−^ was enhanced by 136.9% in BB52, yet, in ZH13, the efflux was deduced 64.1% on the contrary (Figure 4(c)). The behavior of the ion fluxes in mature seedlings has similar characteristic (Figure 4(d)). The wild soybean also represented stronger efflux of Na^+^ and Cl^−^ and influx of H^+^, and weaker K^+^ drain. Compared to young seedlings, the difference was smaller.

## 4. Discussions

### 4.1. The Water Potential and RWC Were Reduced Obviously in Both Types of Soybean and the Cultivated One Was Shown to Be More Sensitive

The osmotic stress is the first effect produced by salt stress. Generally, it starts immediately when the salt concentration exceeds the threshold level [5]. A vast number of researches on different species such as* R. mucronata, Urochondra setulosa, *tomato, and* S. salsa* have reported that the water potential and osmotic potential of plants decreased with an increase in salinity. In this study, we adopted a series of NaCl treatments ranging from 0 to 300 mM. The results suggested that both the water potential and RWC were decreased with the increase in NaCl concentration in two types of soybean (Figure 3). By comparison, the decline was more serious in leaves. For the mature seedling of cultivated soybean,* Glycine max*, the water potential was reduced 11.2 times and the RWC dropped to 64.2% under 300 mM treatment, demonstrating that they have suffered serious osmotic stress. In addition, all the plants under this stress displayed the symptom of water loss and what is more is that 30% percent became dead after the stress. With regard to the mature seedling of wild soybean,* Glycine soja*, though the water potential and RWC were also declined, the extent was small and few plants turned into the state of water loss.

The young seedlings were also influenced by salt stress; thus, the water potential and RWC were lessened as well, yet the effect was a lesser extent (Figure 3). There is no distinct difference in roots between two species, yet the wild soybean maintained relatively higher water potential in leaves. The young seedlings did not turn into dehydration and wilting, but their growth rates were stunted significantly by high concentration of NaCl. We speculated that the plants can manage to regain the original volume and turgor by adjustment after the period time of the stress, yet, despite this, the growth and elongation rate of the cells were lowered down inevitably [43, 44]. These reductions may be due to the decreasing stomatal conductance, the reduced transpiration, the function of the Casparian band, and the enhanced efficiency of water application [5, 45]. In short, the outcome revealed that the wild soybean may own stronger capabilities to maintain water potential, keep water balance, and keep a normal RWC.

### 4.2. NaCl Stress Enormously Promoted the Accumulation of Proline and Glycine Betaine, Especially for the Mature Seedlings of Wild Soybean under 300 mM Treatment

When subjected to salinity, plants will have to take a series of necessary measures. On the one hand, the plant would lessen stomatal conductance and cut down transpiration to adjust to the osmotic stress; on the other hand, the plants would accumulate a great deal of compatible solutes to lower water potential and reduce water desorption. The proline and glycine betaine were the two most effective compatible solutes, playing important protective roles in abiotic stress [4]. Thus, we chose to survey their behaviors to reveal the resistance of the soybean. Our results suggested that the contents of proline and glycine betaine were almost the same under normal conditions, but the existence of the NaCl led to the variations and differences.

For young seedlings the accumulation of proline ascended rapidly when the concentration of the NaCl attained 100 mM or higher. Under 300 mM, the contents of the proline reached 34.4 and 38.2 times, respectively, compared to the control in cultivated and wild soybean. For mature seedlings, the accumulations were 24.9 and 151.1, respectively (Figures 3(a) and 3(b)), indicating that the adjustment capacity of the wild soybean was more extensive. This may also partially explain the relatively higher water potential of the wild soybean under salt stress. Previous studies especially the researches on the synthesis and degradation of the proline have proved the close relationship between proline and salt resistance, and our result was unanimous [34, 46–59].

Glycine betaine was another important metabolite playing an important role in salt resistance. Our research revealed that the manifestation of the glycine betaine was similar to proline (Figure 3). The content of the glycine betaine in mature seedlings of wild soybean achieved twice as much comparatively (Figure 3); thus the accumulation of glycine betaine testified the better resistance of the wild soybean too. Moreover, there were many other compatible solutes such as sugars, sugar alcohols, quaternary amino acid derivatives, and sulfonium compounds, which were demonstrated to participate in the osmotic stress and play important roles. We may certify the superiority of the wild soybean further by observing and analyzing their behaviors.

### 4.3. The Contents of Na^+^ and Cl^−^ Ascended and the Content of K^+^ Descended with Increasing Salt Concentration and the Tendency Was More Pronounced in the Cultivated Soybean

Due to the high concentration of ions in soil around the roots, the plants toke in and accumulated a great deal of ions in cells unavoidable. Once the ions in the cells exceeded a certain amount, the homeostasis of intracellular ion concentrations was disturbed and ionic stress emerged. It was reported that, for the species such as soybean, citrus, and grapevine, Cl^−^ was more toxic through disrupting photosynthesis [51]. Now, ions exclusion from cells and compartmentation into vacuole were the key protective ways adopted by plants [12]. Na^+^ and Cl^−^ were of course the main toxic ions under salinity. K^+^ was proved to take part in enzymatic reactions, protein synthesis, and ribosome functions, and, moreover, Na^+^ could act as an antagonist in absorption and transportation of K^+^ [15]. Thus, we discussed the changes of the contents of Na^+^, K^+^, Cl^−^, and K^+^/Na^+^ ratio and the conclusions were coinciding with the foregoing results. From Figure 3, we can see that the contents of Na^+^ and Cl^−^ were increased obviously by NaCl stress, but the K^+^ was lessened. For the young seedlings, the increase of the Na^+^ and Cl^−^ concentrated mainly on leaves, while the decrease of the K^+^ was due to the change in roots, reflecting their different regulating mechanism. The changes of the K^+^/Na^+^ ratio in leaves were more severe than roots, especially for the roots of wild soybean, and the changing is tiny. In addition, the K^+^/Na^+^ ratio of wild soybean exceeded cultivated soybean under all the treatments and in both root and leaf. In mature seedlings, the situation was almost the same. Notably, the advantage of wild soybean was not as distinct as young seedlings. In general, compared with cultivated soybean, the amplitude of variation of the ion contents in wild soybean was more gentle and smaller, suggesting that the wild soybean had better resistance.

### 4.4. The NMT Revealed the Real-Time Ion Fluxes and Suggested That the Wild Soybean Had Quicker Na^+^ and Cl^−^ Efflux and H^+^ Influx, As Well As Slower K^+^ Efflux

Compared to the ion levels changing, the NMT gave us a more ocular and quicker detection, and the wild soybean exerted more outstanding superiority. Both in young and in mature seedlings, the Na^+^ and Cl^−^ were ejected to the extracellular more quickly, and the H^+^ was imported into cells faster in wild soybean (Figure 4). The K^+^ on the other hand was discharged from cells more slowly, reflecting that the wild soybean had better abilities to maintain K^+^ (Figure 4). These results were coinciding with the emulative absorptions of K^+^ and Na^+^ [15]. Unlike animal, the plant cells make use of the H^+^-ATPases not the Na^+^-ATPases, which they do not have, to create a proton-motive force to drive the afflux of the Na^+^. Therefore, the enhanced H^+^ influx in wild soybean in fact was an indirect reflection of its salt resistance. Under 300 mM NaCl in specific, the flux of H^+^ in cultivated soybean almost arrived at 0 (Figure 4) reflecting that the stress has produced serious damage, while the flux in wild soybean still maintained a steady rising trend to the contrary. The conditions of Cl^−^ were the same. So we can realize that the wild soybean had better salt tolerance, and the capability was more high light under high salt concentration. With the increasing H^+^ entering the cells, more and more Na^+^ was transferred out of the cells by H^+^/Na^+^ at the same time, and the theory had already been elaborated in poplar [52]. In addition, it has been reported that the activity of the plasma membrane (PM) Na^+^/H^+^ antiporters can be induced by NaCl, and many related researches on transporters such as SOS1 and NHXs have also acquired similar conclusions [53–63]. For the mature seedlings, there was the same conclusion. The most obvious differences between them still emerged on 300 mM. Nonetheless, the gap was not as large as young seedlings, especially for the flux of K^+^ [59–63].

## 5. Conclusions

We described here the changes of some indexes which can reflect the influences of salt stress. By comparing the differences between wild and cultivated soybean with the same treatment, we found that the wild soybean (BB52) performed better. The wild soybean displayed higher compatible solutes accumulation, RWC, and K^+^/Na^+^ ratio compared to the cultivated soybean (ZH13). Therefore, the wild soybean as the wild relatives of cultivated soybean can act as a donor for salt tolerance trait [57]. In addition, the wild soybean has been discovered to have more genetic diversity by previous studies too. For example, Luo et al. put forward that the wild soybean employed different tolerance mechanisms from the cultivated soybean [58]. They are all consistent with our results suggesting that the wild soybean was entrusted with better resistance and it may act as a source for us to improve the resistance of the cultivated one or even crops either through conventional breeding or through genetic engineering breeding. Hence, the significance of our study was, on the one hand, to compare the different tolerance levels of soybeans and, on the other hand, to look for potential donors for future breeding.

## References

[1] Ashraf M (2009). Biotechnological approach of improving plant salt tolerance using antioxidants as markers. *Biotechnology Advances*.

[2] Zhu J-K (2001). Plant salt tolerance. *Trends in Plant Science*.

[3] Allakhverdiev SI, Sakamoto A, Nishiyama Y, Inaba M, Murata N (2000). Ionic and osmotic effects of NaCl-induced inactivation of photosystems I and II in Synechococcus sp. *Plant Physiology*.

[4] Ashraf M, Foolad MR (2007). Roles of glycine betaine and proline in improving plant abiotic stress resistance. *Environmental and Experimental Botany*.

[5] Munns R, Tester M (2008). Mechanisms of salinity tolerance. *Annual Review of Plant Biology*.

[6] Yan K, Shao H, Shao C (2013). Physiological adaptive mechanisms of plants grown in saline soil and implications for sustainable saline agriculture in coastal zone. *Acta Physiologiae Plantarum*.

[7] Parida AK, Das AB (2005). Salt tolerance and salinity effects on plants: a review. *Ecotoxicology and Environmental Safety*.

[8] Fricke W, Akhiyarova G, Veselov D, Kudoyarova G (2004). Rapid and tissue-specific changes in ABA and in growth rate in response to salinity in barley leaves. *Journal of Experimental Botany*.

[9] Aziz I, Khan MA (2001). Effect of seawater on the growth, ion content and water potential of Rhizophora mucronata Lam. *Journal of Plant Research*.

[10] Gulzar S, Khan MA, Ungar IA (2003). Salt tolerance of a coastal salt marsh grass. *Communications in Soil Science and Plant Analysis*.

[11] Lu C, Qiu N, Lu Q, Wang B, Kuang T (2002). Does salt stress lead to increased susceptibility of photosystem II to photoinhibition and changes in photosynthetic pigment composition in halophyte Suaeda salsa grown outdoors?. *Plant Science*.

[12] Agarwal PK, Shukla PS, Gupta K, Jha B (2013). Bioengineering for salinity tolerance in plants: state of the art. *Molecular Biotechnology*.

[13] Ashraf M, Akram NA (2009). Improving salinity tolerance of plants through conventional breeding and genetic engineering: an analytical comparison. *Biotechnology Advances*.

[14] Apse MP, Blumwald E (2007). Na^+^ transport in plants. *FEBS Letters*.

[15] Zhu J-K (2003). Regulation of ion homeostasis under salt stress. *Current Opinion in Plant Biology*.

[16] Slabu C, Zörb C, Steffens D, Schubert S (2009). Is salt stress of faba bean (Vicia faba) caused by Na^+^ or Cl^−^ toxicity?. *Journal of Plant Nutrition and Soil Science*.

[17] Bose J, Shabala L, Pottosin I (2014). Kinetics of xylem loading, membrane potential maintenance, and sensitivity of K^+^-permeable channels to reactive oxygen species: physiological traits that differentiate salinity tolerance between pea and barley. *Plant, Cell & Environment*.

[18] Palmgren MG, Nissen P (2011). P-Type ATPases. *Annual Review of Biophysics*.

[19] Mahajan S, Tuteja N (2005). Cold, salinity and drought stresses: an overview. *Archives of Biochemistry and Biophysics*.

[20] Bayuelo-Jiménez JS, Jasso-Plata N, Ochoa I (2012). Growth and physiological responses of phaseolus species to salinity stress. *International Journal of Agronomy*.

[21] Maliro MF, McNeil D, Redden B, Kollmorgen JF, Pittock C (2008). Sampling strategies and screening of chickpea (*Cicer arietinum* L.) germplasm for salt tolerance. *Genetic Resources and Crop Evolution*.

[22] Amirjani MR (2010). Effect of salinity stress on growth, mineral composition, proline content, antioxidant enzymes of soybean. *American Journal of Plant Physiology*.

[23] Ma H, Song L, Shu Y (2012). Comparative proteomic analysis of seedling leaves of different salt tolerant soybean genotypes. *Journal of Proteomics*.

[24] Gao S-Q, Chen M, Xu Z-S (2011). The soybean GmbZIP1 transcription factor enhances multiple abiotic stress tolerances in transgenic plants. *Plant Molecular Biology*.

[25] Phang TH, Shao G, Lam HM (2008). Salt tolerance in soybean. *Journal of Integrative Plant Biology*.

[26] Fan X-D, Wang J-Q, Yang N (2013). Gene expression profiling of soybean leaves and roots under salt, saline-alkali and drought stress by high-throughput Illumina sequencing. *Gene*.

[27] Lauchli A (1984). Salt exclusion: an adaptation of legumes for crops and pastures under saline conditions. *Salinity Tolerance in Plants: Strategies for Crop Improvement*.

[28] Chen H, He H, Yu D (2011). Overexpression of a novel soybean gene modulating Na^+^ and K^+^ transport enhances salt tolerance in transgenic tobacco plants. *Physiologia Plantarum*.

[29] Flowers TJ (2004). Improving crop salt tolerance. *Journal of Experimental Botany*.

[30] Lee J-D, Shannon JG, Vuong TD, Nguyen HT (2009). Inheritance of salt tolerance in wild soybean (*Glycine soja* Sieb. and Zucc.) accession PI483463. *Journal of Heredity*.

[31] Wang K-J, Li X-H (2012). Phylogenetic relationships, interspecific hybridization and origin of some rare characters of wild soybean in the subgenus Glycine soja in China. *Genetic Resources and Crop Evolution*.

[32] Upadhyaya H, Reddy K, Singh S, Gowda C (2013). Phenotypic diversity in *Cajanus* species and identification of promising sources for agronomic traits and seed protein content. *Genetic Resources and Crop Evolution*.

[33] Mallikarjuna N, Senthilvel S, Hoisington D (2011). Development of new sources of tetraploid Arachis to broaden the genetic base of cultivated groundnut (Arachis hypogaea L.). *Genetic Resources and Crop Evolution*.

[34] Wang K, Liu Y, Dong K (2011). The effect of NaCl on proline metabolism in *Saussurea amara* seedlings. *African Journal of Biotechnology*.

[35] Concibido VC, La Vallee B, Mclaird P (2003). Introgression of a quantitative trait locus for yield from *Glycine soja* into commercial soybean cultivars. *Theoretical and Applied Genetics*.

[36] Tuyen DD, Lal SK, Xu DH (2010). Identification of a major QTL allele from wild soybean (*Glycine soja* Sieb. & Zucc.) for increasing alkaline salt tolerance in soybean. *Theoretical and Applied Genetics*.

[37] Wang K-J, Li X-H (2014). Synchronous evidence from both phenotypic and molecular signatures for the natural occurrence of sympatric hybridization between cultivated soybean (*Glycine max*) and its wild progenitor (*G. soja*). *Genetic Resources and Crop Evolution*.

[38] Flexas J, Ribas-Carbó M, Bota J (2006). Decreased Rubisco activity during water stress is not induced by decreased relative water content but related to conditions of low stomatal conductance and chloroplast CO2 concentration. *New Phytologist*.

[39] Bates LS, Waldren RP, Teare ID (1973). Rapid determination of free proline for water-stress studies. *Plant and Soil*.

[40] Gorham J, McDonnell E, Wyn Jones RG (1982). Determination of betaines as ultraviolet-absorbing esters. *Analytica Chimica Acta C*.

[41] Yan K, Chen P, Shao H, Zhang L, Xu G (2011). Effects of short-term high temperature on photosynthesis and photosystem II performance in sorghum. *Journal of Agronomy and Crop Science*.

[42] Maggio A, Miyazaki S, Veronese P (2002). Does proline accumulation play an active role in stress-induced growth reduction?. *Plant Journal*.

[43] Cramer GR (2002). Response of abscisic acid mutants of Arabidopsis to salinity. *Functional Plant Biology*.

[44] Fricke W, Peters WS (2002). The biophysics of leaf growth in salt-stressed barley. A study at the cell level. *Plant Physiology*.

[45] Momayezi M, Zaharah A, Hanafi M (2012). The effects of cation ratios on root lamella suberization in rice (*Oryza sativa* L.) with contrasting salt tolerance. *International Journal of Agronomy*.

[46] Kumar V, Shriram V, Kishor PBK, Jawali N, Shitole MG (2010). Enhanced proline accumulation and salt stress tolerance of transgenic indica rice by over-expressing P5CSF129A gene. *Plant Biotechnology Reports*.

[47] Huang Z, Zhao L, Chen D (2013). Salt stress encourages proline accumulation by regulating proline biosynthesis and degradation in Jerusalem Artichoke plantlets. *PLoS ONE*.

[48] Vendruscolo ECG, Schuster I, Pileggi M (2007). Stress-induced synthesis of proline confers tolerance to water deficit in transgenic wheat. *Journal of Plant Physiology*.

[49] Yamchi A, Rastgar Jazii F, Mousavi A, Karkhane AA, Renu R (2007). Proline accumulation in transgenic tobacco as a result of expression of Arabidopsis Δ1-Pyrroline-5-carboxylate synthetase (P5CS) during osmotic stress. *Journal of Plant Biochemistry and Biotechnology*.

[50] Silva-Ortega CO, Ochoa-Alfaro AE, Reyes-Agüero JA, Aguado-Santacruz GA, Jiménez-Bremont JF (2008). Salt stress increases the expression of p5cs gene and induces proline accumulation in cactus pear. *Plant Physiology and Biochemistry*.

[51] Brini F, Masmoudi K (2012). Ion transporters and abiotic stress tolerance in plants. *ISRN Molecular Biology*.

[52] Sun J, Chen S, Dai S (2009). NaCl-induced alternations of cellular and tissue ion fluxes in roots of salt-resistant and salt-sensitive poplar species. *Plant Physiology*.

[53] Qiu QS, Barkla BJ, Vera-Estrella R, Zhu J, Schumaker KS (2003). Na^+^/H^+^ exchange activity in the plasma membrane of Arabidopsis. *Plant Physiology*.

[54] Martínez-Atienza J, Jiang X, Garciadeblas B (2007). Conservation of the salt overly sensitive pathway in rice. *Plant Physiology*.

[55] Ji H, Pardo JM, Batelli G, Van Oosten MJ, Bressan RA, Li X (2013). The Salt Overly Sensitive (SOS) pathway: established and emerging roles. *Molecular Plant*.

[56] Jiang X, Leidi EO, Pardo JM (2010). How do vacuolar NHX exchangers function in plant salt tolerance?. *Plant Signaling and Behavior*.

[57] Badridze G, Weidner A, Asch F, Börner A (2009). Variation in salt tolerance within a Georgian wheat germplasm collection. *Genetic Resources and Crop Evolution*.

[58] Luo Q, Yu B, Liu Y (2005). Differential sensitivity to chloride and sodium ions in seedlings of Glycine max and G. soja under NaCl stress. *Journal of Plant Physiology*.

[59] Ruan CJ, Xu XX, Shao HB, Jaleel CA (2010). Germplasm-regression-combined (GRC) marker-trait association identification in plant breeding: a challenge for plant biotechnological breeding under soil water deficit conditions. *Critical Reviews in Biotechnology*.

[60] Yan K, Chen P, Shao HB, Shao CY, Zhao SJ, Brestic M (2013). Dissection of photosynthetic electron transport process in sweet sorghum under heat stress. *PLoS ONE*.

[61] Yan K, Chen P, Shao HB (2012). Responses of photosynthesis and photosystem II to higher temperature and salt stress in sorghum. *Journal of Agronomy and Crop Science*.

[62] Yan K, Chen P, Shao HB, Zhao SJ (2013). Characterization of photosynthetic electron transport chain in bioenergy crop Jerusalem artichoke (Helianthus tuberosus L.) under heat stress for sustainable cultivation. *Industrial Crops and Products*.

[63] Shao HB, Chu LY (2013). Some progress in the study of plant-soil interactions in China. *Plant Biosystems*.

